# Cross-sectoral rehabilitation intervention for patients with intermittent claudication versus usual care for patients in non-operative management - the CIPIC Rehab Study: study protocol for a randomised controlled trial

**DOI:** 10.1186/s13063-019-4032-x

**Published:** 2020-01-21

**Authors:** Maj Siercke, Lise Pyndt Jørgensen, Malene Missel, Lau Caspar Thygesen, Pernille Peppercorn Blach, Henrik Sillesen, Selina Kikkenborg Berg

**Affiliations:** 1 Department of Vascular Surgery, Rigshospitalet, Copenhagen University Hospital, Blegdamsvej 9, 2100 Copenhagen, Denmark; 2 Institute of Clinical Medicine, University of Copenhagen, Copenhagen, Denmark; 3 Department of Pathology, Herlev Hospital, Copenhagen University Hospital, Copenhagen, Denmark; 4 Department of Cardiothoracic Surgery, Rigshospitalet, Copenhagen University Hospital, Copenhagen, Denmark; 5 National Institute of Public Health, University of Southern Denmark, Copenhagen, Denmark; 6 Healthcare Center Albertslund, Capital Region of Denmark, Albertslund, Denmark; 7 The Heart Centre, Rigshospitalet, Copenhagen University Hospital, Copenhagen, Denmark

**Keywords:** Intermittent claudication, Cross-sectoral rehabilitation, Physical exercise

## Abstract

**Introduction:**

Intermittent claudication (IC) caused by peripheral artery disease (PAD) is a common cardiovascular disease. Patients with IC have reduced walking capacity, restricted activity levels and mobility, and reduced health-related quality of life. The disease leads to social isolation, the risk of cardiovascular morbidity, and mortality. Non-operative management of IC requires exercise therapy and studies show that supervised exercise training is more effective than unsupervised training, yet many patients with IC lack motivation for changes in health behaviour.

No studies investigating the effects of existing cardiac rehabilitation targeted patients with IC have been published. The aim of this article is to present the rationale and design of the CIPIC Rehab Study, which examines the effect of a cross-sectoral rehabilitation programme versus usual care for patients in non-operative management for IC.

**Methods and analysis:**

A randomised clinical trial aims to investigate whether cardiac rehabilitation for patients with IC in non-operative management versus usual care is superior to treatment as usual. The trial will allocate 118 patients, with a 1:1 individual randomisation to either the intervention or control group.

The primary outcome is maximal walking distance measured by the standardised treadmill walking test. The secondary outcome is pain-free walking distance measured by the standardised treadmill walking test, healthy diet measured by a fat-fish-fruit-green score, and level of physical activity measured by an activity score within official recommendations. Statistical analyses will be blinded.

Several exploratory analyses will be performed. A mixed-method design is used to evaluate qualitative and quantitative findings. A qualitative and a survey-based complementary study will be undertaken to investigate patients’ post-discharge experiences. A qualitative post-intervention study will explore experiences of participation in rehabilitation.

**Discussion:**

The study is the first to assess the effect of a cardiac rehabilitation programme designed for patients with IC. The study will describe how to monitor and improve rehabilitation programmes for patients with IC in a real-world setting. Mixed-method strategies can allow for both exploration and generalisation in the same study, but the research design is a complex intervention and any effects found cannot be awarded a specific component.

**Trial Registration:**

Retrospectively registered in Clinicaltrials.gov identifier: NCT03730623.

## Background

Peripheral artery disease (PAD) is a chronic occlusive arterial disease caused by progressive atherosclerosis [1]. The most common symptom is intermittent claudication (IC), defined as a cramping leg pain that occurs during walking and is relieved by a short period of rest. IC affects 2% of the population (age 50–60) and increases with age to 6–7% (age 65–75) of the population in Western Europe and the USA [2]. Patients with IC have diminished walking capacity, restricted activity levels and mobility, and reduced health-related quality of life [3, 4]. It leads to social isolation and, unless health behaviour and relevant medications are prescribed, it may lead to worsening of disease with the risk of atherosclerotic complications and death [1, 3–8]. Motivation is an important but neglected factor as studies indicate that many patients with IC are not motivated for health behaviour changes in accordance with current recommendations [6, 7, 9]. Due to the risk of complications and limited patency of revascularisation (depending on procedure and anatomic location), current guidelines recommend that patients not requiring surgical revascularisation due to critical limp ischaemia be managed conservatively without surgical intervention [10]. Current practice for managing IC in Danish hospital settings involves brief advice to ‘stop smoking and keep walking’, combined with preventive medications including cholesterol-lowering treatment with statins and antiplatelet therapy [10, 11]. Non-adherence to these recommendations increases the risk of progression from IC to critical ischaemia and limb amputation [1]. It also produces a substantial economic burden on society due to reduced working ability, hospitalisation, and associated personal and social consequences for the individual patient [12, 13]. A recent Cochrane review [14] concluded that for patients with IC exercise is important regardless of whether treatment is revascularisation or overall conservative management. Supervised exercise training (SET) programmes are effective for alleviating symptoms, increasing walking distance, reducing cardiovascular risk factors and improving quality of life. Additionally, SET is relatively inexpensive and cost-efficient compared with other more invasive therapies [13–16]. Although evidence for SET is strong, studies exploring the effects of cross-sectoral rehabilitation intervention on patients treated for IC are lacking. IC rehabilitation is still poorly implemented and knowledge about how to set up an effective programme in a community-based setting is poor [17, 18].

### Rehabilitation

Secondary prevention initiatives including rehabilitation for patients with PAD are recommended in current guidelines [5, 19]. Community-based supervised exercise appears to be at least as effective as exercise programmes provided in hospital settings [20]. Importantly, a study recently reported that attending a hospital-based supervised exercise programme was difficult for patients due to time spent on transportation and logistics [21]. This indicates that intervention in the local community improves patients’ motivation and adherence [22]. Therefore, given the evidence for the beneficial effect of supervised exercise training for patients after acute coronary syndrome, the hypothesis is that patients with IC could also benefit with regard to maximal walking distance (MWD), pain-free walking distance (PWD), health-related quality of life, and physical function. Patients' perspectives on participating in the intervention could shed light on the factors that facilitate or hinder exercise and recommended health behaviour. Knowledge of this may increase both the quality and patient adherence to the conservative management of IC, thereby attenuating the burden of disease and improving quality of life for patients with IC.

## Study objectives

The objectives of the trial are to investigate the effects of a cross-sectoral exercise and lifestyle intervention based on the established rehabilitation programme for patients with ischaemic heart disease versus usual care without rehabilitation in patients with IC. The primary hypothesis is that, compared with the control group, a specialised rehabilitation programme for the intervention group improves MWD in the treadmill walking test after the completed intervention. The three secondary hypotheses are that PWD, diet and level of physical activity improve in the intervention group compared with the control group after 6 and 12 months. Exploratory analyses will test the hypothesis that IC rehabilitation improves quality of life, health behaviour, physical activity and reduces anxiety and depression after 6 and 12 months. The effects, benefits, and motivational factors of conservative management will be examined and patient experiences of the intervention, including factors that support or hinder adherence to the intervention explored.

## Design

The CIPIC Rehab Study is designed to develop evidence-based knowledge on rehabilitation among patients with IC. It is a cross-sectoral, multidisciplinary, randomised clinical trial designed to examine the effects of an IC rehabilitation programme compared with usual care for patients in non-operative treatment for IC. Accordingly, the trial combines quantitative and qualitative research methods. The mixed methods are integrated by applying the explanatory sequential design [23, 24]. The rationale for this approach is that the quantitative findings provide a general understanding of the research problem through statistical results, and qualitative findings refine and explain the results by exploring participants’ views in greater detail. Qualitative research coupled with randomised controlled trials can contribute to developing and evaluating complex healthcare interventions; it may be particularly useful in evaluating interventions that involve social and behavioural processes that are difficult to explore or capture using quantitative methods alone [25, 26]. A pragmatic world view is the philosophy underpinning the study [23].

### Study population and eligibility criteria

Consecutive patients at the Department of Vascular Surgery at the Rigshospitalet in Copenhagen, Denmark will be screened for inclusion and approached for study participation.

Inclusion criteria are: patients with newly diagnosed IC treated conservatively; age > 18 years; speak and understand Danish; able to provide informed written content; citizens of the eight municipalities of Greater Copenhagen belonging to the Healthcare Centre; and able to perform physical exercise. Exclusion criteria are: failure to understand and cooperate according to the trial instructions; co-morbidity complicating physical activity and exercise training, and lack of informed content.

### Study procedure

When the informed content is signed, baseline data will be collected including a questionnaire administrated by the primary investigator. After baseline data collection, randomisation is conducted. Computer-generated block randomisation in four blocks has been done by an independent statistician and delivered in envelopes blinded from investigators. Randomisation is conducted by ongoing inclusion numbers marked on the envelopes.

### Control group – usual care

Patients randomised to the control group will initially receive the department’s usual, brief advice about exercise therapy (walking), smoking cessation, and preventive medical treatment with antiplatelet therapy and statins. The IC patients will receive written information about medication, walking exercise, and a logbook for self-reporting of walking behaviour in the outpatient clinic at the Department of Vascular Surgery, Rigshospitalet. Patients in the control group will follow standard follow-up procedure for patients treated for IC.

### Experimental intervention group

The intervention group initially receives the usual care in the outpatient clinic at the Department of Vascular Surgery; additionally, patients’ home communities offer courses in smoking cessation. Patients will receive a pedometer and be asked to self-report walking behaviour and steps in a logbook. The patient brings the logbook to the consultation with the physiotherapist, who initiates the startup training, supplies the motivation and explains the goal for the physical activity. Patients in the intervention group will follow the specialised cardiac rehabilitation programme for patients with IC. The intervention is based on experiences of cardiac rehabilitation and guidelines of the Danish National Board of Health and European Society of Cardiology [19]. Theories about personalised feedback and self-efficacy will be used as a method for encouraging behavioural changes to improve health outcomes [27].

### Physical exercise training component

Training sessions will take place at a Healthcare Centre within the municipality of Greater Copenhagen. The main goal of the exercise is to improve the patient’s physical capacity and health behaviour, such that this subsequently results in physical and psychological health benefits. Supervised exercise training is also targeted, relieving the fear and uncertainty the patient may feel towards physical activity. Two specialised cardiac rehabilitation physiotherapists with specific insight into IC will plan and supervise participants’ exercise. This entails patients actively engaging, in groups with up to ten in 24 supervised physical exercise sessions, each lasting one hour with two weekly sessions. The exercises include varied forms of physical exercise all combined to accommodate the patients’ own goals regarding walking distance. The physiotherapists will administer and record a six-minute walking test and 30-second chair stand test prior to and at completion of the intervention. Pedometer and self-reported walking behaviour are a part of the consultation used to increase or sustain daily physical exercise at least 30 minutes per day. The results will be used as part of an individual motivational interview with each patient after completion of the 24 training sessions.

### Supervised exercise training programme

The exercise training protocol will consist of a 10–15-minute warm-up, followed up by a 45–50-minute combination of strength and circuit training. The exercise training programme is based on national guidelines for cardiac rehabilitation [28]. The warm-up will be based on either bicycling, with a focus on using the forefoot when pedalling, or walking in different variations, i.e. walking on toes, heels, walking sideways, walking lunges and walking at different paces. In strengthening the large muscle groups, there will be a primary focus on the leg muscles. The strength exercises for the upper body will primarily be performed as an intermission, in the exercise for the lower body. Different exercise equipment will be used to create resistance in the exercise training, i.e. elastic bands, body bars, dumb bells, and strength-training machines. The exercise will vary from 1 × 15, 2 × 15, and 3 × 10 repetitions, based on low to moderate intensity of 40–60% of the maximum muscle strength [28]. The circuit training will primarily be based on activity for the lower limbs, i.e. walking and running at different paces and variations, walking combined with an exercise, i.e. high knee lifts, kick backs, calf raises, and different relay races in teams. The circuit training will also involve interval training, of varying lengths, depending on both the different exercises and the patients’ individual limits due to lower limb pain. Two of the sessions will be based on using and practising pole striding at a nearby outdoor training park. In addition to the physical activity component, the programme will also contain components of health education for improving the self-efficacy of physical activity in the patients and therefore seek to affect health behaviour. Five of the sessions will contain 10–15 minutes of health education, which will include the use of tools developed by Steno Diabetes Center, Copenhagen, Denmark. These tools were developed for supporting patients in making long-term health-related changes, and for the use of health professionals in health education for patients with chronic illness [29]. Furthermore, the health education will contain motivational, group-based dialogue with the patients about their health behaviour and ability to participate in physical activity in their own neighbourhood. There will also be motivational conversations concerning the patients’ daily use of and achievements with the pedometer and logbook handed out to each patient at the start of the intervention.

### Education in groups and individual consultation

The aim of the intervention is to provide emotional support, improve coping skills, and to respond to physical symptoms. Education and information about the disease prepares the patient for expected symptoms and sensations, and dialogue and shared reflections facilitate strategies for coping with symptoms and experiences associated with the condition, for example when leg pain is part of the treatment for getting better. The group education is a two-hour-long session, about the pathophysiology of IC, medications, health behaviour, disease management, quality of life, and coping with the disease. The principal investigator (MS), who is an experienced cardiac rehabilitation nurse with specific knowledge of IC to ensure protocol compliance, will perform the intervention. Information given will also be based on national guidelines and standard treatment of patients with IC. A clinical dietician will advise participants in a two-hour-long group session about healthy diet and atherosclerosis, and in addition give access to individual consultation.

Albert Bandura’s Social Cognitive-Behavioral Theory and self-efficacy inspires the intervention. Its focus is on the dynamic interaction of person and behaviour; the individual’s actual ability to perform the appropriate behaviour; learning a new skill or knowledge by observing others; external responses to the individual’s behaviour that either encourage or discourage the behaviour expectations: the anticipated consequences of a behaviour; and self-efficacy: the person’s confidence in his or her ability to perform a behaviour [27, 30]. Consequently, the individual, the group, spouses, and surroundings in a rehabilitation setting are important. Spouses are therefore invited to participate in group sessions as well as in individual sessions.

Studies show that text messages can facilitate lifestyle changes [31–33]. After completion of exercise training, participants are offered personalised motivational follow-up text messages. The content, frequency, and duration of the text messages are agreed upon individually for the next 8 months and will be reassessed at follow-ups after 3 and 6 months.

### Outcomes and data collection

**Fig. 1.**
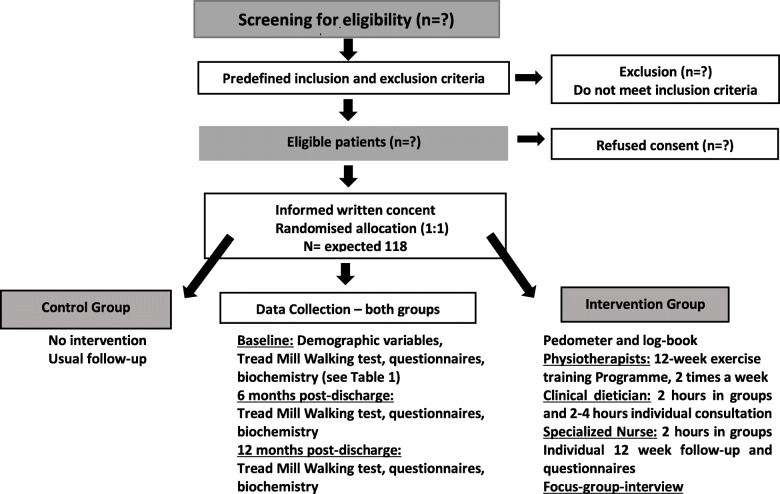
Patient flow

Data will be collected at admission, discharge, 6 months and 12 months administrated by the primary investigator (see Table 1). The primary and the secondary outcomes reflect the primary modifiable factors of the intervention, and a number of explorative outcomes will be collected to evaluate the effect and meaning of the intervention (see Table 2). The post-discharge experiences of patients in the intervention group will be explored through semi-structured qualitative interviews. Patient flow is illustrated in Fig. 1.

**Table 1:** The CIPIC Rehab Study - exploratory quantities subjected to post hoc analysis

Quantity	Time of measure	Type of quantity
Demographic		
Sex	Baseline	Binary (M/F)
Age, height, weight, body mass index (BMI)	Baseline	Continuous
Marital, occupational, educational status	Baseline	Categorical
Clinical		
Charlson Comorbidity Index [34]	Baseline, 6, 12	Categorical
Hypertension	Baseline 6, 12	Binary (Y/N)
Smoking+ Fagerströms test, (Alcohol Timeline Followback)	Baseline, 6, 12	Categorical
Medication (routine drugs; antiplatelet; statins and other medication)	Baseline, 6, 12	Categorical
Nutritional screening ‘HjerteKost’: fat-fish-fruit-green score [35]	Baseline, 6, 12	Categorical
Paraclinical		
Blood work (biomarkers, cholesterol, HBa1C, Hg, thyroid)	Baseline, 6, 12	Continuous
Physical function		
The standardised treadmill walking test [36, 37]	Baseline, 6, 12	Continuous
Six-minute walking test (before and after supervised exercise training) [38]	Baseline, 3	Continuous
Sit to stand test (before and after supervised exercise training) [39]	Baseline, 3	Continuous
Level of physical activity (0–7 times a week)	Baseline, 6, 12	Categorical
Questionnaires		
HADS, Hospital Anxiety and Depression Scale [40]	Baseline, 6, 12	Categorical
VascuQol, Vascular Quality of Life questionnaire [41]	Baseline, 6, 12	Categorical
PAM13, 13-item Patient Activation Measure [42]	Baseline, 6, 12	Categorical
Pedometer, text message (intervention group)	3 months, 6	Binary (Y/N)
Participation in dietician and nurse session (intervention group)	3 months	Binary (Y/N)

*M/F* male/female, *Y/N* yes/no

**Table 2:** Focus group - interview topics

Supervised exercise training: physiotherapist. Content and education	
Education session: nurse and dietician. Content and education	
Patients’ experiences of participating in the intervention group.	
Knowledge and uncertainty about IC	
Experiences of factors and barrier that supported or hindered adherence to the intervention.	
Factors that influence coping strategy, persistent lifestyle changes	
Importance of environment and togetherness with similar patients	
Empathy, support and motivation	
Risk factor management	
Coping behaviours	
Change interventions	
Attitudes, beliefs, how to handle the pain	
Feeling better mentally	
Accessibility and compliance	
Self-monitoring goal setting	
Exercise logbook and pedometers. Motivational text message.	
Specific walking advice to promote self-managed walking	
Quality of life	
Solution behaviour change techniques	
Patient satisfaction of participate in the IC rehabilitation programme and point out if any suggestions for changes.

### Primary outcome

MWD will be measured by the standardised treadmill walking test based on a graded protocol (3.2 km/hour with 2% increase every 2 minutes). Treadmill assessment has the highest reliability when using a graded protocol together with outcome measurements, such as initial claudication distance and absolute claudication distance. Results of treadmill testing are expressed as the initial claudication distance, the moment claudication pain begins and the absolute claudication distance, the moment the test has to stop due to the maximal level of bearable claudication pain [36, 37]. The follow-up treadmill walking test will be performed by a research assistant blinded as to the patients’ group affiliations.

### Secondary outcome

PWD will be measured by the standardised treadmill walking test as described above and with a numeric rating scale for pain [37, 43]. Daily physical activity is measured by self-reported number of times per week of walking or physical exercise activity of at least 30 minutes, as recommended by the National Board of Health [44]. Diet will be measured by a diet questionnaire (HjerteKost), a validated Danish instrument with 19 items. The scale offers two scores, a fat and a fish-fruit-green score, each of which can range from 0 to 18. To be able to achieve the term ‘healthy’ the score must be at least 75% in both the fat and the green scores [35]. The instrument is validated and recommended by the National Board of Health [28].

### Exploratory outcomes

Smoking is measured by self-reported smoking behaviour by the Fagerström Test for Nicotine Dependence. The self-administered questionnaire has good internal validity and a good correlation with nicotine levels as an instrument for measuring addiction to tobacco [45]. Alcohol consumption will be measured by the Alcohol Timeline Followback (TLFB). TLFB has been shown to be a psychometrically sound assessment instrument for obtaining retrospective daily estimates of alcohol consumption. TLFB has been extensively evaluated in various settings, over varying reporting intervals and with diverse drinker populations and has been found to have very good measurement properties [46, 47].

### The Hospital Anxiety and Depression Scale (HADS)

HADS is a 14-item instrument that measures symptoms of anxiety (HADS-A) and depression (HADS-D). The scale offers two subscales, each of which can range from 0 to 21. Scores of 0–7 for either subscale are regarded as normal; 8–10 suggest the presence of a mood disorder; and 11 and above suggest the probable presence of a mood disorder. This tool has been translated and validated in many countries and its capacity to detect anxiety and depressive disorders is widely recognised [48].

### The Vascular Quality of Life questionnaire (VascuQoL)

VascuQoL (VQ6) is a PAD-specific instrument recommended as one of the preferred questionnaires when evaluating quality of life outcomes in patients with PAD. The VQ6 is a six-item questionnaire, developed using a combination of qualitative and quantitative methodology. The VQ6 has acceptable to good psychometric properties regarding data quality, scale assumptions, targeting, validity and reliability. Further, VQ6 seems to be easy to use and comprehend within the target population of patients with PAD [41].

### The Patient Activation Measure (PAM)

PAM-13 is a 13-item instrument for evaluating educational interventions aimed at improving patient engagement. Patient activation specifies the level of patients’ engagement and may contribute to better self-management, higher engagement in treatment and greater patient satisfaction. The European translations of PAM-13 resulted in four instruments with good psychometric capabilities for measuring patient activation. All items have five possible responses with scores ranging from 0 to 4: (1) disagree strongly, (2) disagree, (3) agree, (4) agree strongly or (0) not applicable [42, 49].

## Complementary studies

Numerous data will be collected to evaluate the effect and meaning of the intervention.

### Quantitative data

The quantitative study consists of an individual questionnaire survey conducted as interview by the principal investigator. The survey including data about feasibility: participation (number of times), use of pedometer (yes/no), logbook (yes/no) and to what extent it has motivated daily physical exercise, exercise choice after the course, and text messages (yes/no). Results from the physiotherapist six-minute walking test and 30-second chair stand test, before and after in metres/number are also included.

### Qualitative explorative data

As a part of the study, brief individual interviews exploring course satisfaction, suggestions for changes, and the relevancy of the various rehabilitation components will be conducted. Furthermore, focus group interviews of patients participating in the intervention group will also be conducted. Prior to interviewing, an interview guide will be developed. It will be used to help explore patient experiences of training and teaching sessions, factors helping or hindering improvement in health behaviour, the use of the pedometer, logbook, and text message influenced motivation/adherence, patient satisfaction with the intervention and suggestions for future rehabilitation programmes. Research questions will be developed using knowledge from existing qualitative studies in the field and the individual brief interviews [6, 7, 9, 15, 17, 50, 51] (see Table 2). The focus group interviews will be conducted by the principal investigator (MS) and two assistant moderators that register key points and takes field notes [52]. Patients will be recruited during their 3- and 6-month follow-ups at the Healthcare Centre or at the Department of Vascular Surgery as a convenience sampling with consecutive recruitment of participants according to the groups in which they exercised during the training sessions. To embrace the potential impact of any team spirit developed during the training session, we consider focus groups and recruitment of participants according to training groups to be relevant. The interviews will be held in well-known surroundings in the Healthcare Centre. The size of the focus groups will be five to eight participants to secure an opportunity for each person to share insights, experiences and observations. Smaller groups allow more in-depth conversation and afford each person a greater opportunity to speak. ‘Information power’ will guide the adequate sample size and the number of focus group interviews [52].

### Data collection and data analysis

The interviews will be audio recorded and transcribed verbatim. Interviews are anticipated to last approximately one hour. Thematic analysis according to Braun & Clarke will be used to analyse data [53]. This means combining a coding analysis with the content of the focus group discussion [11, 13]. Derivation of themes will be identified by an exploratory analysis to present selected patterns relevant for the study aim and collected data. Numbers of data coders, description of the coding tree, software program, illustrated themes/findings, quotation identification, consistency between the data presented and the findings, as well as the clarity of major and minor findings will be a part of the analysis [54, 55]. The thematic analysis will be used as a systematic approach to the analysis of quality data from the focus group interviews. That involves identifying themes or patterns of meaning by coding and classifying data textually, according to themes and interpreting the resulting thematic structures by seeking commonalties, relationships, overarching patterns, theoretical constructs, or explanatory principles [56].

## Statistical analysis

A trial-independent statistician will make a blind analysis of the data and the primary and secondary analyses will be performed according to the intention-to-treat principle. We will use general regression models for the continuous outcomes and logistic regression models for binary outcomes. In the analysis of the primary outcome, the outcome (MWD at 6 months) will be analysed with adjustment for baseline MWD, sex or age (included continuously). For the three secondary outcomes – PWD distance, level of physical activity and diet at 6 months – the analysis will be done similarly with adjustment for baseline values, sex and age (continuous). As exploratory analyses of MWD, PWD, smoking behaviour, diet and patient-related outcome measures, mixed general and generalised models with repeated measurements will be used including measurements at baseline, 6 and 12 months in the same model. These models will also be used for all other explorative outcomes. In these models, the interaction between intervention group and time is of primary interest, indicating different developments after intervention start. In the case of significant results in the primary outcome, sensitivity analyses will be performed to estimate the potential effect of data missing at random by a worst-case scenario. Let X be the group where a beneficial effect is observed, and Y be the other group. Missing values in group X will be imputed by the minimum value found in the material and missing values in group Y will be imputed by the maximum value found. The primary outcome will be tested first using a significance level of 0.05. Analyses of the secondary and exploratory outcome measures as planned above will be analyzed with no *p* value adjustment due to multiplicity. Instead, the interpretation of these results will be assessed in the light of multiple testing, i.e. statistically significant effects will be interpreted in the context of increased risk of type I error. The clinical effect size will be reported by Cohen’s d. Per protocol analyses of the primary and secondary outcomes will be performed.

### Sample size and power calculation

The expected average baseline value of MWD has been set to 120 m with a detected 50% improvement (60 m). There is a wide variance in MWD in this patient group and consequently the standard deviation (SD) is set at 100 m, based on an expected improvement in walking ability of approximately 50% to 200% [18]. With a 5% significance level and 80% power, it will thus be necessary to include 88 patients to detect an improvement of 60 m in MWD in the intervention group at the 12-month follow-ups, compared with the control group. Owing to the previously mentioned risk of co-morbidities, combined with an expected drop-out, a drop-out of 25% must be expected, therefore the investigators plan to include 118 patients in total (59 in each group).

## Discussion

This randomised clinical trial is the first to examine the effect of a cross-sectoral exercise and health behaviour intervention based on the established cardiac rehabilitation programme for patients with IC. The CIPIC Rehab Study will provide evidence on the rehabilitation needs of patients treated conservatively for IC, ánd insight into the patient benefits and motivational factors of conservative management experiences of the intervention. The results can be used to make recommendations for a specialised IC rehabilitation programme, which healthcare professionals and policymakers may use to make qualified, evidence-based decisions in everyday clinical practice and as a foundation for national and international guidelines. With a positive outcome, some of the possible effects could be lower morbidity and a decrease in the use of the public health system. This is advantageous for both patients and society. Whether it produces neutral, negative or positive results the study will have implications for clinical practice and follow-up care for patients treated for IC. The study has been designed to meet the criteria for high quality in non-pharmacological randomised clinical trials [57] with central randomisation, blinded assessment of the exercise outcome, and blinded analysis by a study-independent statistician. Detailed information on the intervention received and usual care will be collected, including self-initiated exercise training during the trial period. The secondary outcomes of self-rated mental health are subjective by nature [58–60]. The trial is designed with multiple statistical comparisons, therefore results of the explorative analyses will be interpreted with caution.

### Trial status

Recruitment began on 1 April 2017 and end of the 12-month follow-up of all patients will be completed in april 2020 in accordance to protocol number: H-17004183/clinicaltrials.gov.

Inclusion was initiated on 5 December 2017 and completed in 28 June 2019. End of the 12-month follow-up of all patients will be completed at the end of June 2020. The results of the trial and complementary studies will be published in relevant international peer-reviewed journals. Authorship will be determined according to the guidelines of the International Committee of Medical Journal Editors.

## Supplementary information

**Additional file 1.** SPIRIT 2013 checklist.

## Data Availability

Not applicable.

## Abbreviations

HADS: Hospital Anxiety and Depression Scale
IC: Intermittent claudication
M/F: Male/Female
MWD: Maximal walking distance
PAD: Peripheral artery disease
PAM13: Patient Activation Measure
PWD: Pain-free walking distance
SET: Supervised exercise training
TLFB: Alcohol Timeline Followback
VascuQol: Vascular Quality of Life questionnaire
