# Supplementary material for: Cross-sectoral rehabilitation intervention for patients with intermittent claudication versus usual care for patients in non-operative management - the CIPIC Rehab Study: study protocol for a randomised controlled trial
Source: Trials. 2020 Jan 21;21:105. doi: 10.1186/s13063-019-4032-x (PMC6975054; doi:10.1186/s13063-019-4032-x)
Supplement: Supplementary file 1 — Additional file 1. SPIRIT 2013 checklist. [file 13063_2019_4032_MOESM1_ESM.doc]

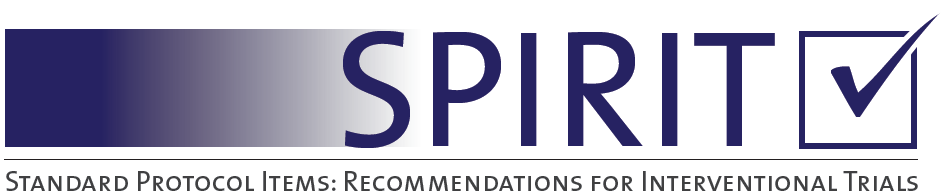


SPIRIT 2013 Checklist: Recommended items to address in a clinical trial protocol and related documents*

| Section/item | ItemNo | A randomised clinical trial of cross-sectoral rehabilitation intervention for patients with intermittent claudication versus usual care for patients in non-operative management - The CIPIC Rehab study |
| --- | --- | --- |
| **Administrative information** | | |
| Title | 1 | Descriptive title identifying the study design, population, interventions, and, if applicable, trial acronym |
| Trial registration | 3 | Trial identifier and registry name. If not yet registered, name of intended registry |
|  | 3 | All items from the World Health Organization Trial Registration Data Set |
| Protocol version | 3 | Date and version identifier |
| Funding | 18 | Sources and types of financial, material, and other support |
| Roles and responsibilities | 18 | Names, affiliations, and roles of protocol contributors |
|  | 18 | Name and contact information for the trial sponsor |
|  | 18 | Role of study sponsor and funders, if any, in study design; collection, management, analysis, and interpretation of data; writing of the report; and the decision to submit the report for publication, including whether they will have ultimate authority over any of these activities |
|  | 18 | Composition, roles, and responsibilities of the coordinating centre, steering committee, endpoint adjudication committee, data management team, and other individuals or groups overseeing the trial, if applicable (see Item 21a for data monitoring committee) |
| Introduction |  |  |
| Background and rationale | 3 | Description of research question and justification for undertaking the trial, including summary of relevant studies (published and unpublished) examining benefits and harms for each intervention |
|  | 5 | Explanation for choice of comparators |
| Objectives | 5 | Specific objectives or hypotheses |
| Trial design | 5 | Description of trial design including type of trial (eg, parallel group, crossover, factorial, single group), allocation ratio, and framework (eg, superiority, equivalence, noninferiority, exploratory) |
| Methods: Participants, interventions, and outcomes | | |
| Study setting | 6 | Description of study settings (eg, community clinic, academic hospital) and list of countries where data will be collected. Reference to where list of study sites can be obtained |
| Eligibility criteria | 6 | Inclusion and exclusion criteria for participants. If applicable, eligibility criteria for study centres and individuals who will perform the interventions (eg, surgeons, psychotherapists) |
| Interventions | 7 | Interventions for each group with sufficient detail to allow replication, including how and when they will be administered |
|  | 6 | Criteria for discontinuing or modifying allocated interventions for a given trial participant (eg, drug dose change in response to harms, participant request, or improving/worsening disease) |
|  | 7 | Strategies to improve adherence to intervention protocols, and any procedures for monitoring adherence (eg, drug tablet return, laboratory tests) |
|  | - | Relevant concomitant care and interventions that are permitted or prohibited during the trial |
| Outcomes | 10 | Primary, secondary, and other outcomes, including the specific measurement variable (eg, systolic blood pressure), analysis metric (eg, change from baseline, final value, time to event), method of aggregation (eg, median, proportion), and time point for each outcome. Explanation of the clinical relevance of chosen efficacy and harm outcomes is strongly recommended |
| Participant timeline | 18 | Time schedule of enrolment, interventions (including any run-ins and washouts), assessments, and visits for participants. A schematic diagram is highly recommended (see Figure) |
| Sample size | 16 | Estimated number of participants needed to achieve study objectives and how it was determined, including clinical and statistical assumptions supporting any sample size calculations |
| Recruitment | 6 | Strategies for achieving adequate participant enrolment to reach target sample size |
|  | | |
|  |  |  |

| STUDY PERIOD | Enrolment | Allocation | Post Allocation | Close out |
| --- | --- | --- | --- | --- |
| Time point | April 2017 | Dec 2017 | May 2018 | April 2020 |
| Eligibility screen | x | x | x |  |
| Informed content |  | x | x |  |
| Allocation | x | x | x |  |
| Intervention/control group |  | x | x | x |
| Assessment |  | x | x |  |
| Demographic  Sex  Age, height, weight, Body Mass Index (BMI)  Marital, occupational, educational status  Clinical  Charlson Comorbidity Index  Hypertension  Smoking+ Fagerströms test, alcohol (Time-Line-Follow Back)  Medication  Nutritional screening ‘HjerteKost’: fat-fish-fruit-green score  Paraclinical  Blood work (biomarkers, cholesterol, HBa1C, Hg, thyroid)  Physical function  The standardized Treadmill Walking Test  Six min walking test (Before and after Supervised exercise training)  Sit to stand test (Before and after supervised exercise training)  Level of physical activity (0-7 times a week)  Questionnaires  HADS, Hospital Anxiety and Depression Scale(40)  VascuQol, The Vascu -Quality of Life questionnaire  PAM13, The Patient Activation Measure |  | X (baseline) | X (6 months) | X (12 months) |
| Pedometer,  Text message (intervention group)  Participation in dietician and nurse session (intervention group) |  |  | 3 months |  |
| Outcome variables:  Maximal walking distance.  Pain-free walking distance.  Healthy diet  level of physical activity |  | x | x | x |
| Qualitative and a survey-based complementary study. Focus group interview. patients’ post-discharge experiences |  |  | 6 months | August 2019 |
